# Combining machine learning and structure-based approaches to develop oncogene PIM kinase inhibitors

**DOI:** 10.3389/fchem.2023.1137444

**Published:** 2023-03-10

**Authors:** Haifa Almukadi, Gada Ali Jadkarim, Arif Mohammed, Majid Almansouri, Nasreen Sultana, Noor Ahmad Shaik, Babajan Banaganapalli

**Affiliations:** ^1^ Department of Pharmacology and Toxicology, Faculty of Pharmacy, King Abdulaziz University, Jeddah, Saudi Arabia; ^2^ Department of Genetic Medicine, Faculty of Medicine, King Abdulaziz University, Jeddah, Saudi Arabia; ^3^ Department of Biology, College of Science, University of Jeddah, Jeddah, Saudi Arabia; ^4^ Department of Clinical Biochemistry, Faculty of Medicine, King Abdulaziz University, Jeddah, Saudi Arabia; ^5^ Department of Biotechnology, Acharya Nagarjuna University, Guntur, India; ^6^ Princess Al-Jawhara Al-Brahim Center of Excellence in Research of Hereditary Disorders, King Abdulaziz University, Jeddah, Saudi Arabia

**Keywords:** PIM kinase, classification models, virtual screening, molecular docking, cancer drug treatment

## Abstract

**Introduction:** PIM kinases are targets for therapeutic intervention since they are associated with a number of malignancies by boosting cell survival and proliferation. Over the past years, the rate of new PIM inhibitors discovery has increased significantly, however, new generation of potent molecules with the right pharmacologic profiles were in demand that can probably lead to the development of Pim kinase inhibitors that are effective against human cancer.

**Method:** In the current study, a machine learning and structure based approaches were used to generate novel and effective chemical therapeutics for PIM-1 kinase. Four different machine learning methods, namely, support vector machine, random forest, k-nearest neighbour and XGBoost have been used for the development of models. Total, 54 Descriptors have been selected using the Boruta method.

**Results:** SVM, Random Forest and XGBoost shows better performance as compared to k-NN. An ensemble approach was implemented and, finally, four potential molecules (CHEMBL303779, CHEMBL690270, MHC07198, and CHEMBL748285) were found to be effective for the modulation of PIM-1 activity. Molecular docking and molecular dynamic simulation corroborated the potentiality of the selected molecules. The molecular dynamics (MD) simulation study indicated the stability between protein and ligands.

**Discussion:** Our findings suggest that the selected models are robust and can be potentially useful for facilitating the discovery against PIM kinase.

## Introduction

Proto-oncogene PIM-1 kinase is a member of the serine/threonine protein kinase family (Narlik-Grassow et al., 2014). PIM kinases are involved in cancer cell survival, proliferation, and tumor growth and are overexpressed in a number of hematological malignancies, in addition to solid cancers such as pancreatic, prostate, and colon cancers (Amson et al., 1989; Li et al., 2006; Nawijn et al., 2011). PIM-1, PIM-2, and PIM-3 are the three highly homologous genes that make up the PIM family. This kinase family is highly homologous with the kinase domains, especially in the linker region and the ATP-binding sites (Warfel and Kraft, 2015). These enzymes are constitutively expressed in tumors and are becoming more widely acknowledged as crucial survival signal mediators in malignancies, stress responses, and neurological development. PIM-1 kinase is a genuine oncogene that is the focus of drug development research initiatives since it has been linked to the emergence of leukemias, lymphomas, and prostate cancer (Li et al., 2011; Le et al., 2015; Huang et al., 2022). PIM kinases regulate the network of signaling pathways that are critical for tumorigenesis and development, making them attractive drug targets (Drygin et al., 2012; Tursynbay et al., 2016).

The crystal structure of PIM-1 has been published by numerous independent groups in both the presence and the absence of its inhibitors (Wang et al., 2013; Nonga et al., 2021). Structural research on PIM-1 has found a number of distinctive characteristics that set it apart from other kinases with known structures. The catalytic domain of PIM-1 kinase spans amino acid positions 38 to 290 and includes a conserved glycine loop motif at positions 45 to 50, phosphate-binding sites at positions 44 to 52 and 67, and a proton acceptor site at position 167. The hunt for small-molecule ATP-competitive inhibitors with the potential to develop into novel targeted oncology treatments has been sparked by the involvement of the PIM kinases in important cancer hallmarks. The majority of PIM-1 inhibitors have failed to evolve into a new anticancer medication despite having excellent biochemical potency, largely because they were found to have subpar pharmacological qualities (Dakin et al., 2012; Drygin et al., 2012; Ogawa et al., 2012; Vivek et al., 2017; Zhao et al., 2017; Park et al., 2021). Due to their therapeutic value in cancer, the discovery of PIM-1 inhibitors has increasingly attracted much attention in past few years. The rate of new PIM inhibitor discovery has increased significantly, and there has been demand for a new generation of potent molecules with the right pharmacologic profiles that can probably lead to the development of PIM kinase inhibitors that are effective against human cancer.

This work was undertaken to develop machine learning-based classification models to identify a new class of PIM-1 inhibitors. Under this approach, four different machine learning methods were applied to develop the classification models. These models were further used to screen chemical libraries to retrieve novel potent PIM-1 inhibitors. In addition, we also carried out molecular docking and molecular dynamics simulations to investigate the interaction and stability within the catalytic site of PIM-1 kinase. This multistage approach allows us to screen large chemical libraries efficiently and effectively in a reasonable time. Moreover, it can also help us identify novel chemical scaffolds for potent PIM-1 inhibitors.

## Materials and methods

### Data collection and model building

All chemical compounds with activity against PIM-1 were collected from the literature and the ChEMBL database (Gaulton et al., 2012). Inorganic and duplicate compounds were removed from the list. Generally, compounds with IC_50_ ≤ 10 μM will likely be “active,” predicting a large number of active molecules. However, such a high fraction of active compounds cannot be expected from any experimental platform. Therefore, in order to make the most efficient use of costly experimental validation, the optimal model should identify compounds with affinity higher than 10 μM. The higher the value, the higher the drug dose needed to achieve the required potency and, thus, the higher the chance of “off-target” activity. To address this issue, we chose to set the decision boundary at IC_50_ ≤ 1 μM for active molecules. Molecular descriptors were calculated using the PaDEL software (Yap, 2011). A two-tier selection procedure was applied to select the best descriptors. First, we randomly selected one descriptor from a pair showing >0.85 correlation. Second, descriptors were reduced using the Boruta method (Kursa et al., 2010). We used four different machine learning methods, namely, Support Vector Machine (SVM) (Mitchell, 1997), random forest (Breiman, 2001), Extreme Gradient Boosting (XGBoost) (Chen and Guestrin, 2016), and kappa nearest neighbor (kNN) (Voulgaris and Magoulas, 2008), to build the classification models. All the classification experiments and calculations were conducted using the R.3.0.2 environment (http://www.R-project.org/) and Python (http://www.python.org/) platform. The compounds used in training and test sets are given in Supplementary Tables S1 and S2, respectively.

### Model validation

A receiver operating characteristic (ROC) plot and area under the curve (AUC) were used to assess the performance of the model (Hanley and McNeil, 1983; Park et al., 2004). In Table 1, the terms precision (Eq. 1), recall (Eq. 2), accuracy (Eq. 3), and F1 score (Eq. 4) are defined along with their relationships to the statistical performance calculations used to assess the quality of the model.

$$Precision=\frac{True\;positive}{True\;positive+False\;Negative},$$ (1)

$$Recall=\frac{True\;positive}{True\;positive+False\;Negative},$$ (2)

$$Accuracy=\frac{TP+TN}{TP+TN+FP+FN},$$ (3)

$$F1=1.\frac{Precision\;X\;Recall}{Precision+Recall}.$$ (4)

**TABLE 1:** Evaluation metrics for the test set.

Method	Descriptors	Precision	Recall	Accuracy (Q)	F1 score	AUC
XGBoost	All descriptors	0.82	0.81	0.83	0.97	0.89
	Boruta	0.81	0.79	0.85	0.80	0.88
	MACCS	0.80	0.76	0.81	0.77	0.92
Random forest	All descriptors	0.85	0.81	0.86	0.98	0.91
	Boruta	0.86	0.81	0.87	0.83	0.92
	MACCS	0.80	0.76	0.82	0.78	0.90
SVM	All descriptors	0.74	0.73	0.78	0.86	0.83
	Boruta	0.75	0.72	0.78	0.71	0.82
	MACCS	0.70	0.73	0.70	0.69	0.82
kNN	All descriptors	0.77	0.75	0.80	0.75	0.84
	Boruta	0.72	0.67	0.75	0.68	0.78
	MACCS	0.81	0.76	0.82	0.77	0.82

### Applicability domain

In order to highlight the region of the chemical space that contains the chemicals for which the model is expected to make accurate predictions, a well-validated predictive model needs to have a defined applicability domain (AD) (Rakhimbekova et al., 2020). Any predictive model must verify its constraints in terms of its structural domain and response space. As a result, determining a model’s AD and evaluating the accuracy of its predictions are both challenging tasks. These QSAR models typically use the training set to cover a certain chemical space. The model’s predictions are accurate if any query compound falls within this definition of AD. If not, the prediction might not conform to the model’s presumptions. Principal component analysis (PCA) (Sushko et al., 2010) has been employed in our work to define the AD of the compounds used in this study.

### Y-randomization

To test the robustness of the proposed models, y-randomization was applied. This technique involves randomly mixing up the values of the target variable in the training set (Rücker et al., 2007; Lipiński and Szurmak, 2017). The same parameters used in the initial model are then applied to a new prediction generated with the scrambled data. Every estimate of the model’s accuracy was recorded. In total, 50% of the compounds in the training set were resampled and used in a 500-run y-randomization test.

### Similarity calculations

The Tanimoto coefficient (Tc) (Eq. 5) was computed using MACCS-166 fingerprints to quantify chemical similarity. The active and inactive chemicals in the training set were compared against false and true positive compounds in systematic pairwise similarity computations.

$$Tc=\frac{C}{A+B-C}.$$ (5)

### Substructure analyses

Molecular substructures related to PIM activity were analyzed using the distribution of MACSS fingerprints in active and inactive compounds (Eq. 6).

$$Frequency=\frac{\sum_{i}^{N}FP\left(1|0\right)}{N}X100.$$ (6)

### Analysis of probability scores

Additionally, the probability scores of the developed classification models were examined. In general, a molecule is defined as inactive if its probability score is lower than 0.5, while a compound with a probability score of 0.5 is considered active (Ponzoni et al., 2019). The more this score approaches 1, the more confident we are in our prediction. Here, we examined the probability score distributions for TP (true positive), TN (true negative), FP (false positive), and FN (false negative) results.

### Chemical database screening

The developed models were used to screen the hits against PIM-1. The NCI library and Maybridge databases were used for virtual screening. The National Cancer Institute maintains a repository of compounds that have been evaluated as potential anticancer agents. These compounds represent unique structural diversity based on synthetic and natural products. The Maybridge library consists of a highly diverse set of over 53,000 lead-like compounds. Maybridge Hit-to-Lead was designed for medicinal chemistry, allowing SAR development and hit-to-lead optimization. The following filters were used to select the hits: Filter 1: compounds predicted to be active by all the validated models; Filter 2: compounds having a probability score; and Filter 3: compounds falling within the chemical space of the training set. These compounds were further processed for molecular docking, followed by molecular dynamics simulations. Finally, compounds with the best affinity and conformance within the active site were selected and analyzed.

### Molecular docking

Molecular docking was implemented to identify the best physical confirmation of inhibitor binding within the active site of PIM-1 kinase. The PIM kinase enzyme structure was taken from the Protein Data Bank (PDB ID: 5KZI). All of the docking simulations for this work were performed using AutoDock Vina (Trott and OlsonAutoDock, 2009) with a 1 spacing, default exhaustiveness, and full ligand flexibility. The grid resolution was internally set to 1Å. We set the number of binding modes to 10 and exhaustiveness to 8. A cubical grid of size 60 × 60 × 60 size with 0.375 Å spacing was used around the active sites of the protein. To acquire the structure in the PDBQT format, polar hydrogen atoms were added using AutoDock Tools 92.

### Molecular dynamics simulations

Selected best compounds were further subjected to molecular dynamics (MD) simulations using Groningen Machine for Chemical Simulations (GROMACS v5.1.5) (Pronk et al., 2013). The parameters and coordinate files for PIM-1 kinase and selected potential hit compounds were generated using the CHARMM27 forcefield in GROMACS and PRODRG, respectively. The TIP3P water model was used for each simulation system, which was neutralized by the addition of Na^+^ ions in a dodecahedron periodic box. Energy minimization was performed for 50,000 nstep using the steepest descent algorithm to avoid steric clashes. Equilibration of each system was performed in two stages: the first phase was carried out with a constant number of particles, volume, and temperature (NVT) ensemble for 500 ps at 300 K, using the V-rescale thermostat (Bussi et al., 2007); and in the second phase, the pressure of each system was equilibrated for 500 ps at a constant number of particles, pressure, and temperature (NPT) at 1 bar using a Parrinello–Rahman barostat (Parrinello and Rahman, 1981). Each equilibrated system was simulated for 30 ns under periodic boundary conditions to avoid edge effects. Electrostatic interactions were handled by the particle mesh Ewald (PME) method, while the heavy-atom bonds were restrained using the LINCS algorithm.

## Results

### Model development and evaluation

**FIGURE 1:**
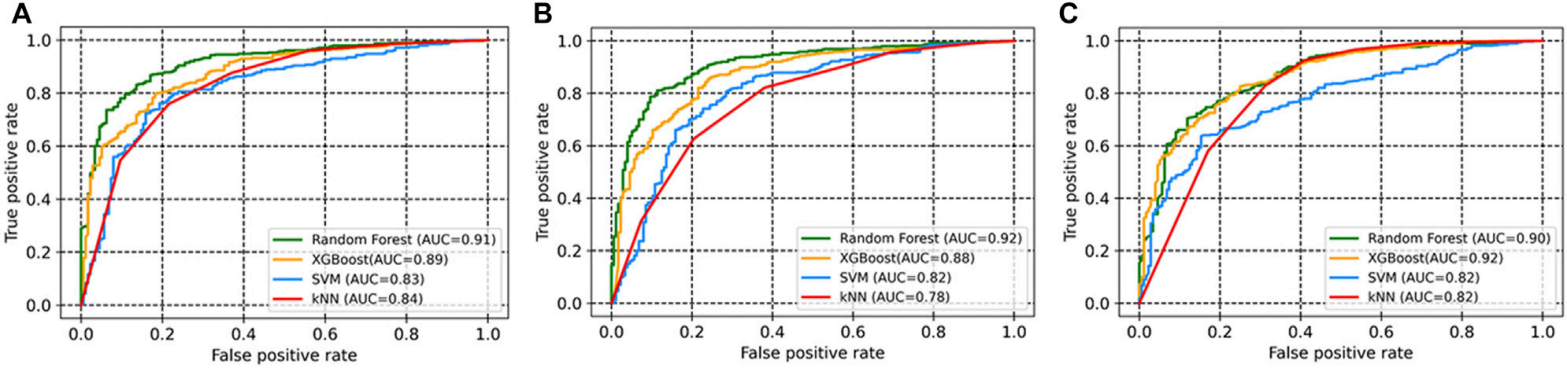
ROC curves of the models based on four machine learning approaches for **(A)** all descriptors; **(B)** selected descriptors (Boruta method); **(C)** MACCS fingerprints.

In total, 54 descriptors from the set of 240 were eventually selected using the Boruta method (Supplementary Table S3). All these descriptors belonged to 12 different classes. The descriptors include autocorrelation, information content, atom-type electrotopological state, Burden modified eigenvalues, molecular distance edge, carbon type, and molecular linear free energy relation*.* The models were trained using four machine learning methods (SVM, random forest, XGBoost, and kNN). Evaluation metrics for the developed models are given in Table 1, including accuracy, recall, precision, F1 Score (a measure of a model’s accuracy, which takes into account both precision and recall), and Area Under the Curve (AUC) values. SVM, random forest, and XGBoost performed than kNN according to these metrics in combination with the selected descriptor set. Among the three, random forest achieved the best accuracy, at 0.87 for the test set (with selected descriptors), as compared to SVM (0.78) and XGBoost (0.84). In addition, these models also had significant AUC values (Figure 1).

### Applicability domain and y-randomization

**FIGURE 2:**
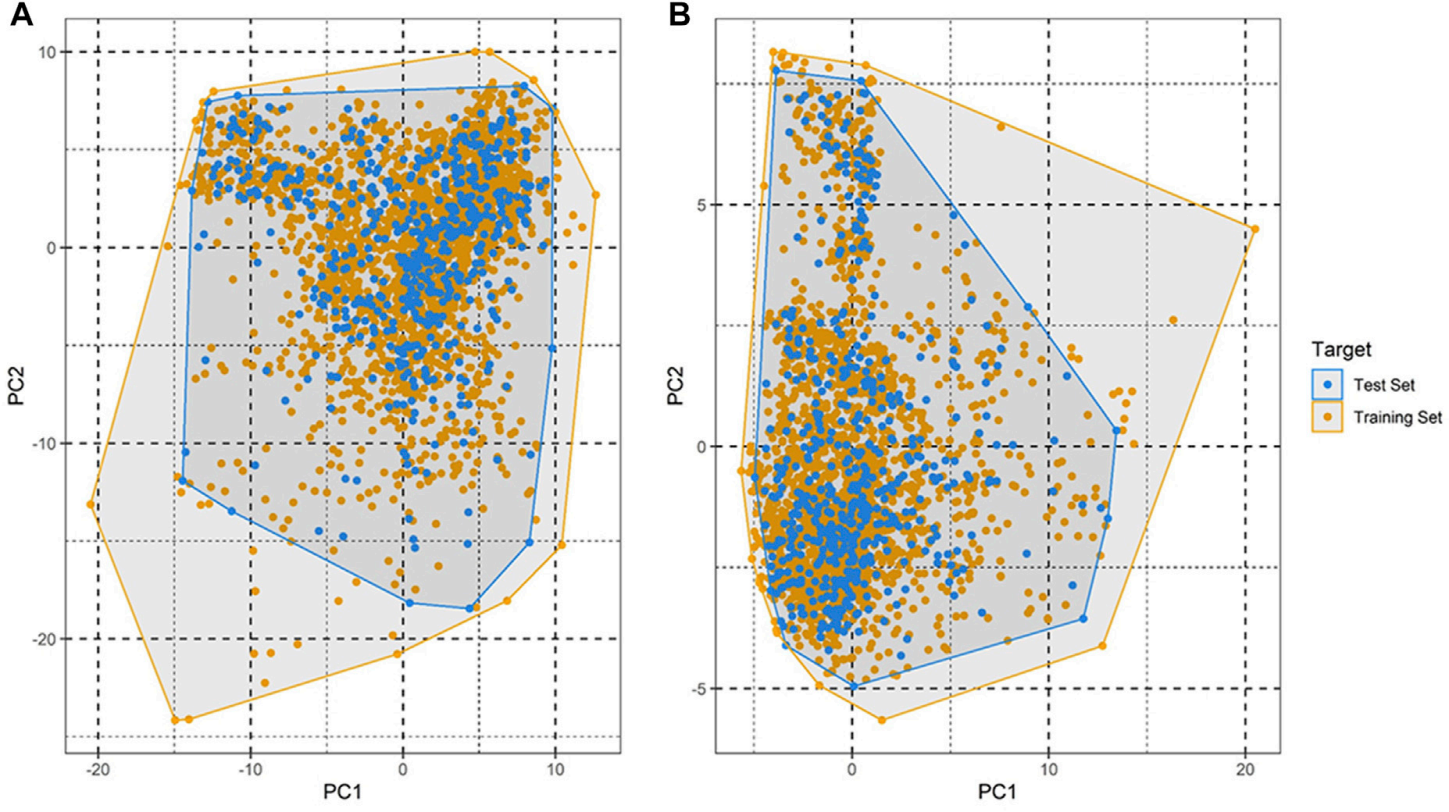
Applicability domain plot based on principal component analysis (PCA) for **(A)** training set and **(B)** test set.

**FIGURE 3:**
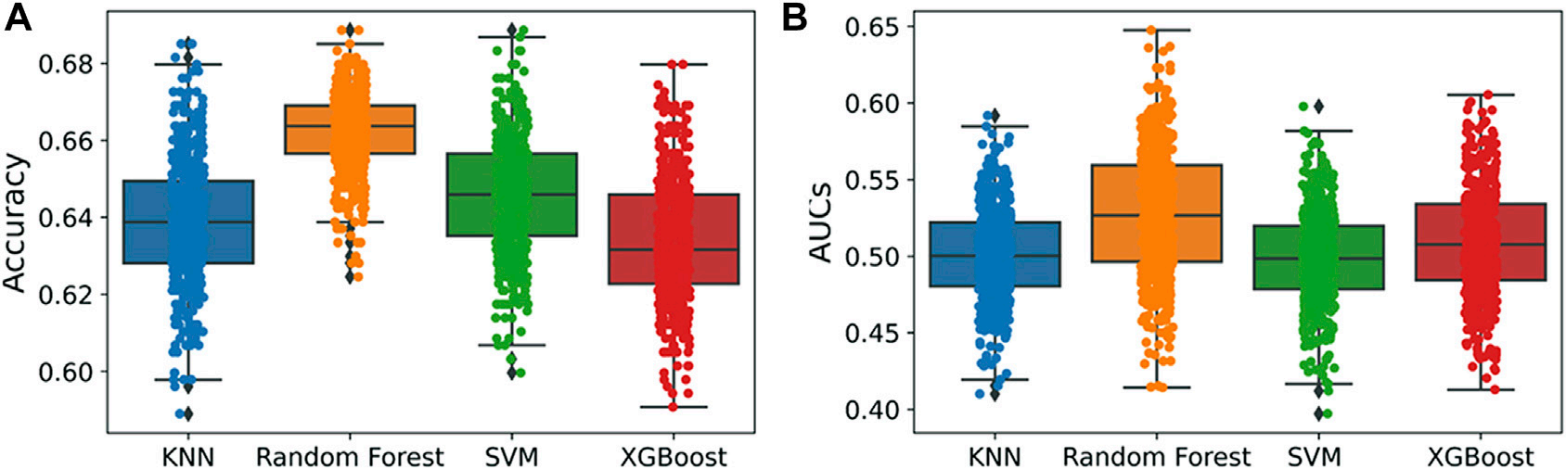
Y-randomization models. **(A)** Accuracy; **(B)** AUC values. A total of 500 y-randomization runs were performed.

An applicability domain (AD) analysis was performed to check the reliability of the generated classification models. Figure 2 shows a scatter plot of the PC1 and PC2 coordinates derived from the set of selected PIM-1 compound descriptors. The training and test compounds share similar PC1 and PC2 coordinates, suggesting that predictions were within the applicability domain (AD) of both the training and test sets. To check the robustness of the developed models, y-randomization tests were performed (Rücker et al., 2007). Y-randomization test accuracies were found to be lower, and none of the random trials achieved higher scores than our main models (Figure 3). The average accuracy across all randomly generated models were found to be less than 0.58. This confirms that the selected models are robust and reliable and were not generated by chance correlations. A pairwise comparison of the compounds in each cluster was found to reflect reasonable Tanimoto coefficient similarities between them.

### Probability analyses

**FIGURE 4:**
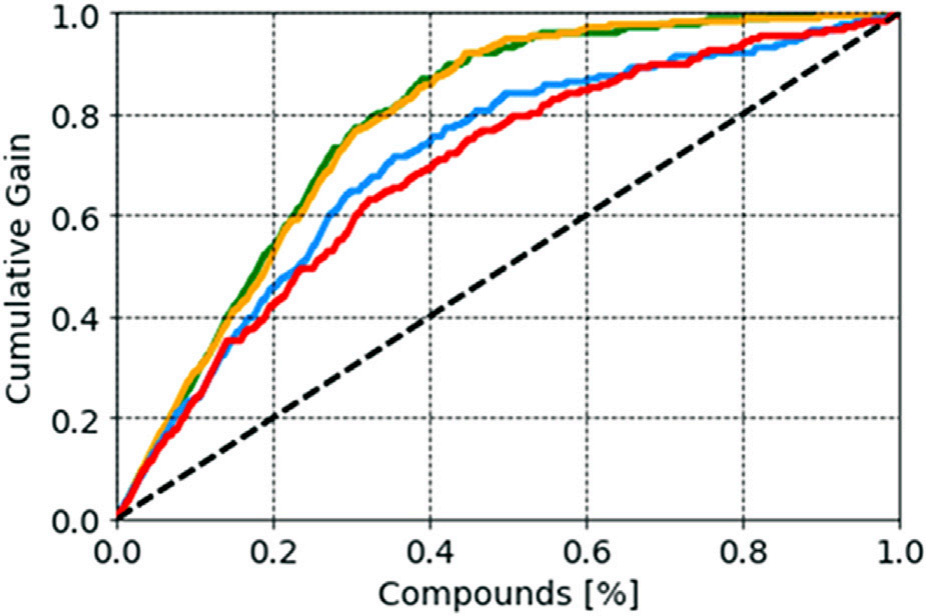
Probabilistic distribution plot showing cumulative gain for the developed models.

Probability scores of the selected models, reflecting the probability of belonging to each class, were also analyzed. It is known that a compound with a probability score of ≥0.5 is classified as active, whereas a molecule with a probability below <0.5 is classified as inactive. As this score approaches 1, the higher the value, the higher the model’s confidence in the prediction is (Minerali et al., 2020; Esposito et al., 2021). In our study, we analyzed the distribution of probability scores among TN (true negative), FP (false positive), TP (true positive), and FN (false negative) results. For the SVM model, compounds with a probability score of more than 0.80 (an average value) were more likely to be active, whereas compounds with a probability score of 0.36 were more likely to be inactive. In the case of the random forest model, a compound with a probability score of more than 0.87 was more likely to be active, whereas a compound with a probability score of 0.24 was more likely to be inactive. Random forest achieved values of 0.95 and 0.11 for active and inactive compounds, respectively, indicating greater success in predicting compound activity with the desired probability score (Supplementary Figure S1). False positive compounds were predicted with probability scores of 0.63, 0.65, and 0.69 for the random forest, XGBoost, and SVM models, respectively. In contrast, false negative compounds were found to have probability scores of 0.31, 0.42, and 0.14 for the random forest, SVM, and XGBoost models, respectively. Each predictive model’s effectiveness in the early recognition of hits was visually evaluated using a cumulative gain plot (Table 2). The cumulative gain curve is an evaluation curve that evaluates the model’s performance and contrasts the outcomes with a random selection. It displays the percentage of targets identified when taking into account a particular portion of the population that has the highest likelihood of being a target based on the model. The comparison showed that the XGBoost and random forest methods performed better than SVM and kNN in terms of early recognition of hits (Figure 4).

**TABLE 2:** Probability scores and docking scores of the selected compounds.

Compound ID	Classifier probability				Binding energy
	XGBoost	Random forest	SVM	kNN	
CHEMBL303779	0.82	0.74	0.84	0.78	−8.34
CHEMBL690270	0.76	0.70	0.92	0.85	−7.56
CHEMBL748285	0.72	0.74	0.68	0.63	−9.78
EBM-MPC	0.81	0.75	0.71	0.71	−8.45

### MACCS fingerprint analyses

Molecular substructures related to the PIM-1 activity of the compounds can be identified by analyzing the bits in the MACCS fingerprints. We analyzed the MACCS fingerprints showing a reasonable difference between active and inactive compounds (Supplementary Table S4). The occurrence of MACCS fingerprints differed significantly between active and inactive compounds in the training dataset, suggesting that the substructures represented by these features may be closely related to PIM-1 activity. Descriptions and the number of occurrences of these substructures are listed in Supplementary Table S4. It was found that MACCS38, MACCS52, MACCS92, MACCS98, MACCS107, MACCSFP142, *etc.* are prevalent in active molecules. This is consistent with previous studies, which shows that compounds with such functional groups have therapeutic potential against PIM kinase (Tsuganezawa et al., 2012; El-Hawary et al., 2018; Park et al., 2021).

### Database screening and molecular interaction analyses

**FIGURE 5:**
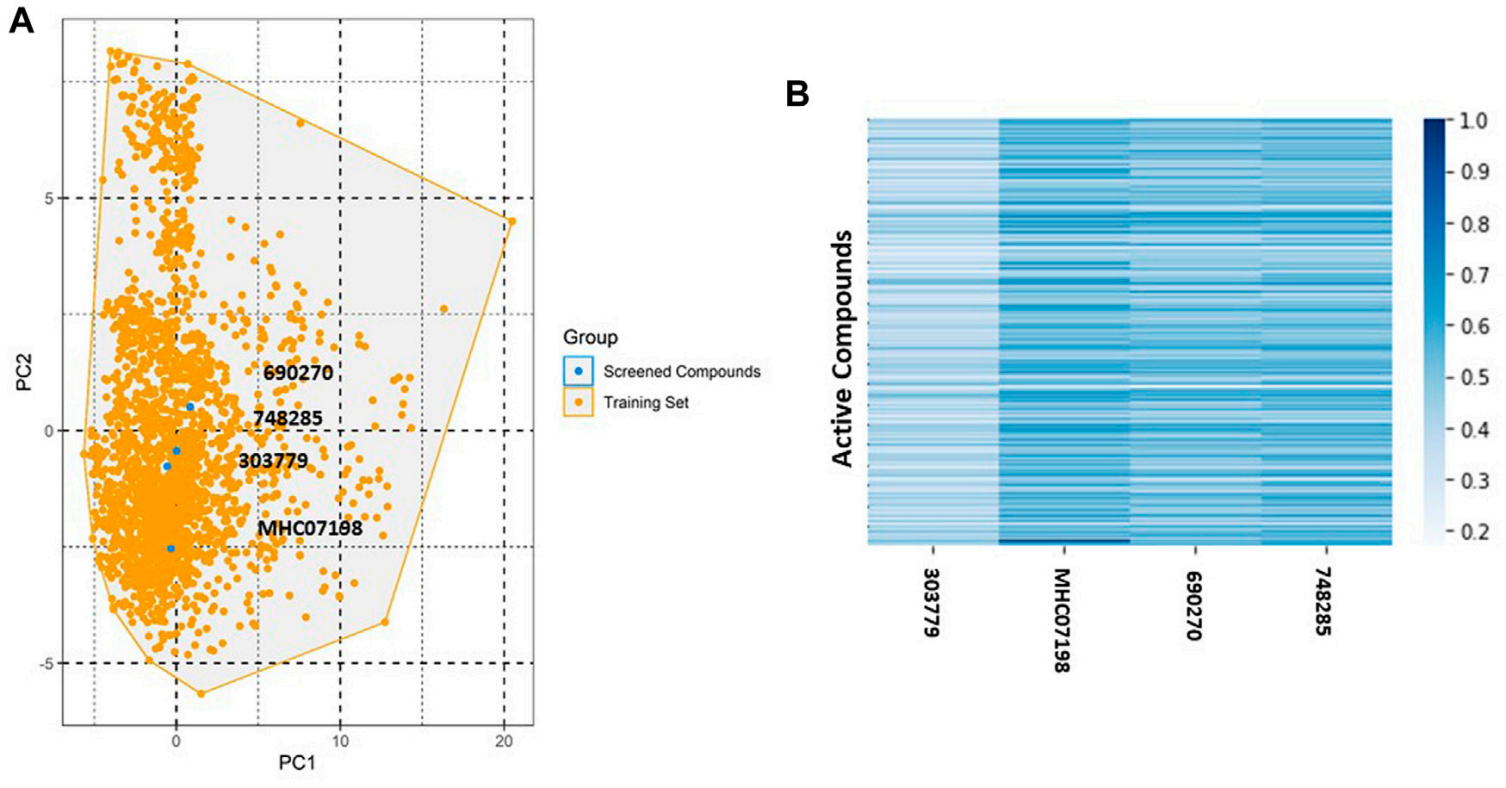
Chemical space and similarity analyses for selected compounds. **(A)** Chemical space of selected compounds; **(B)** heat map of the distance matrix for the selected compounds and active compounds in the training set.

**FIGURE 6:**
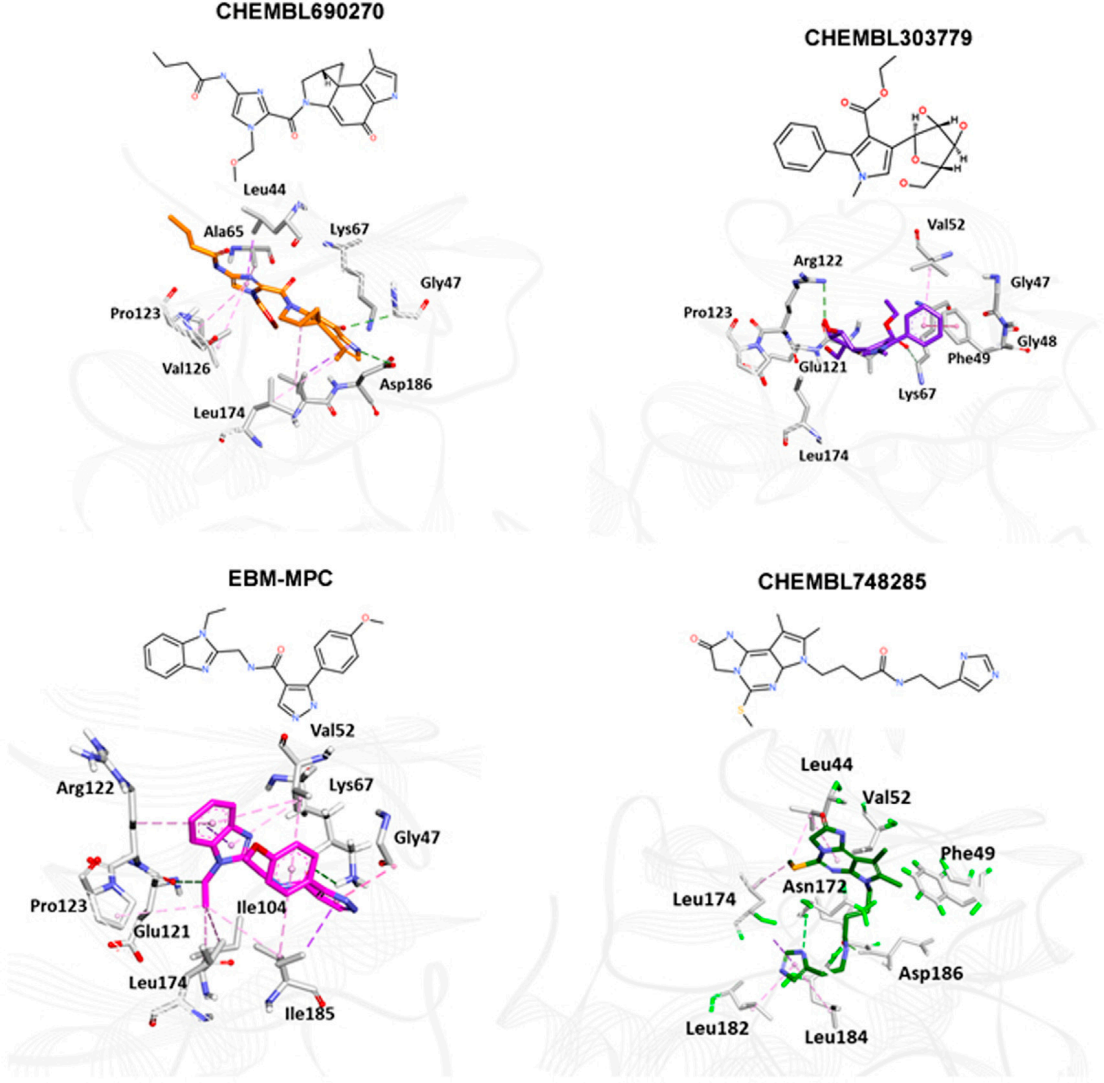
Binding mode analyses of selected compounds within the active site of PIM-1 kinase. Active site residues are shown as gray sticks; the protein backbone is shown as a light gray wire; hydrogen bonds are shown with a green dashed line.

The NCI and Maybridge databases were used to screen the potential hits from validated models. Commonly predicted active compounds with high probability scores were selected and further filtered out within the applicability domain (AD) of the training set. These compounds were further subjected to molecular docking simulation (Table 2). Finally, four compounds (CHEMBL303779, CHEMBL690270, CHEMBL748285, and N-[(1-ethylbenzimidazol-2-yl)methyl]-3-(4-methoxyphenyl)-1H-pyrazole-4-carboxamide (EBM-MPC)) were observed to have reasonable binding affinity and stable interaction with the catalytic residues in the active site (Table.3 and Figure 5). A literature survey revealed that Leu44, Lys67, Glu121, and Asp186 are crucial for the interaction of inhibitors (Tsuganezawa et al., 2012; El-Hawary et al., 2018; Park et al., 2021). It can be observed in Figure 4 that CHEMBL690270, CHEMBL303779, and EBM-MPC form hydrogen bond interactions with Lys67 and hydrophobic interactions with Asp186 (Figure 6). In contrast, CHEMBL748285 forms hydrogen bonds with Asp186 (Figure 6). The quinazoline ring of compounds was involved in multiple p–alkyl interactions. In addition, a number of hydrophobic contacts, particularly residues Leu44, Gly47, Phe49, Ile104, and Leu120, stabilize interaction with hits. PIM inhibitors fall into two broad categories: ATP mimetics, which form hydrogen bonds with the glutamate residue that serves as the hinge (Glu121), and non-ATP mimetics, which bind far from the hinge or interact with the hinge through hydrophobic interactions with a number of residues in the specific hydrophobic pocket that serves as the hinge environment (El-Hawary et al., 2018; Park et al., 2021). The Tanimoto coefficient (Tc) similarity score of these selected hits was found to be ≤ 0.5 with high-activity compounds (Figure 5B).

**TABLE 3:** Binding mode analysis of the four selected inhibitors.

Compound	Hydrogen bonding	Hydrophobic interaction	H-bond range (Å)	Hydrophobic interaction range (Å)
CHEMBL303779	Lys67 and Arg122	Gly45, Gly47, Gly48, Phe49, Ala65, Lys67, Ile104, Leu120, Glu121, Arg122, Pro123, Val126, and Leu174	2.7–3.2	3.3–4.9
CHEMBL690270	Lys67 and Asp186	Leu44, Gly45, Phe49, Lys67, Ile104, Val126, Asp128, Glu171, Asn172, Leu174, and Asp186	2.4–2.6	3.3–4.7
EBM-MPC	Lys67 and Glu121	Gly47, Val52, Lys67, Ile104, Leu120, Glu121, Pro123, Val126, Leu174, and Asp186	2.8–3.0	3.6–4.89
CHEMBL748285	Asn172 and Asp186	Leu44, Val52, Phe49, Asn172, Leu174, Leu182, Leu184, and Asp186	1.6–3.1	3.6–4.4

### MD simulation analyses

**FIGURE 7:**
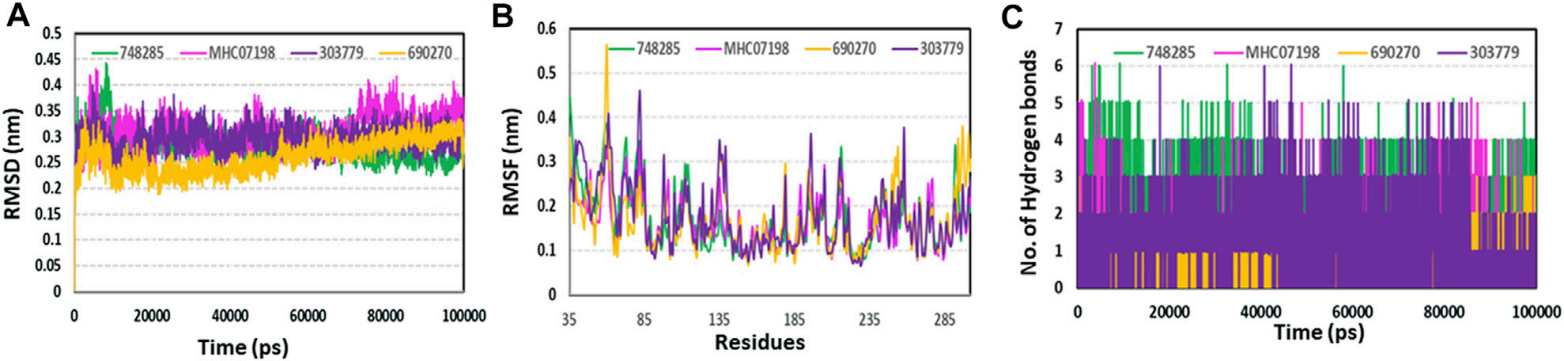
Molecular dynamics simulation analyses. **(A)** RMSD plot; **(B)** RMSF plot; **(C)** hydrogen plot for selected compounds to illustrate protein–ligand stability during a 100-ns simulation.

By analyzing 100-ns MD trajectories, the structural changes to PIM-1 upon inhibitor binding were studied. We examined the RMSD of the protein backbone and the RMSF of the protein’s alpha-carbon atoms. As shown in Figure 6, all the systems exhibited stability throughout the 100-ns simulation. The average RMSD value for all four systems was observed to be below 0.31 nm, which indicated that simulated complexes displayed RMSD values below the threshold. The average RMSD values further showed that the CHEMBL690270 PIM-1 complex displayed less deviation (0.26 nm), whereas CHEMBL303779 and CHEMBL748285 demonstrated similar average values of 0.34 nm (Figure 7A). RMSF is a significant value, used to characterize each residue’s fluctuation rate upon ligand binding. It was observed that the inhibitor binding residues (Leu44, Phe49, Lys67, Glu121, and Asp186) did not fluctuate significantly (Figure 7B).

## Discussion

This study was designed with the aim of building a classification model to predict potential hits for PIM-1 kinase. Four different machine learning approaches were used to build the models. Our proposed models performed well in terms of accuracy, F1 score, precision, and recall. We used the area under the receiver operating characteristic curve approach to compare classifiers. The ROC curve is a graphical representation that contrasts a classifier’s true positive rate and false positive rate at various threshold levels. The area under this curve, or AUC, is thus a useful metric for assessing machine learning algorithms, since it shows the degree of separability (Parrinello and Rahman, 1981). A ROC curve with a higher AUC value implies greater sensitivity in identifying active molecules and specificity in rejecting inactive compounds (Figure 1). In addition, our study also distinguished and ranked the top 18 variables, including 2D autocorrelation, Burden modified eigenvalues, and topological charge*.* These descriptors have the capacity to distinguish between active and inactive compounds.

QSAR Classification models must undergo an extensive validation process, and the reliability of those models must be objectively determined. The OECD guidelines state that a model must have a clearly defined domain of applicability (Dwyer et al., 2013). Additionally, the dataset for such models with a defined AD should cover a broad chemical space and a diverse range of structural types. The AD of PIM kinase inhibitors has been defined using a principal component analysis-based approach for model development. A sufficient level of assurance in the produced models can be seen in the 2D plot obtained from the first two PCs, which represents the training and test set compounds, illustrating their structural variety and similar chemical space (Figure 2). To assess the likelihood of a random correlation for a chosen descriptor, y-randomization was utilized. This technique is used to assess the reliability or robustness of QSAR models and is recognized as one of the most effective validation processes (Rücker et al., 2007). By comparing a developed model’s performance to the average measure of 500 random models, which are obtained by using the same parameters as those used to construct the original model along with a randomly scrambled target variable class, the statistical significance of the developed model can be examined. The results of the y-randomization tests demonstrated that the models created for this study did not exhibit these connections by chance and that a true structure–activity relationship existed (Figure 3).

Fingerprints describe the molecular makeup of a compound. The description of each molecule is given as a string of binary substructures called a fingerprint. The corresponding fingerprint bit is set to 1 if the specified substructure is present in the given molecule; otherwise, it is set to 0. In our study, we used MACCS fingerprints to represent the presence of structures and their representative substructures in active and inactive compounds. These molecules contained MACCS65, MACCS128, and MACCS90. Compounds having such substructures were found to exhibit reasonable levels of activity toward PIM-1 kinase (Akué-Gédu et al., 2010; Dwyer et al., 2013; Hu et al., 2015; Wurz et al., 2015; Li et al., 2016).

To identify potent PIM-1 inhibitors, virtual screening of the NCI and Maybridge databases was performed using the validated models. To gain structural insight relevant to the inhibitory activities of the newly identified inhibitors, their binding modes in the binding site of PIM-1 were examined. Figure 6 shows the most stable binding configurations of selected four compounds derived via docking simulations with potent inhibitors. These compounds appear to be accommodated in a similar way in the binding site of PIM1 (Xia et al., 2009; Abdelaziz et al., 2018; Ibrahim et al., 2022). The necessity of the interactions with the hinge region and Gly-loop residues (Qian et al., 2005; Pogacic et al., 2007; Tsuganezawa et al., 2012; Casuscelli et al., 2013; Fan et al., 2016; Abdelaziz et al., 2018; Bima et al., 2022; Ibrahim et al., 2022; Shaik et al., 2022) for tight binding to PIM-1 was also implicated with potent inhibitors (Xia et al., 2009; Ibrahim et al., 2022). Moreover, these four compounds can also interact with the activation loop including the Asp186 residue. A hydrophobic cavity is formed among the Ala65, Ile104, Phe187, Val52, Lys67, and Leu120 residues, and this maintains molecular stability through various hydrophobic forces. Similar interactions have also been noted in earlier published investigations, highlighting the significance of these amino acids for the assembly of PIM-1 inhibitor complexes (Tsuganezawa et al., 2012; El-Hawary et al., 2018; Park et al., 2021). Residue Lys67 is known to be significant in stabilizing the interaction with the compound and to play an important role in the catalytic activity of PIM-1 (Pogacic et al., 2007; Fan et al., 2016). In our study, we found that all four compounds interacted with Lys67, either with hydrogen bonds or through hydrophobic contact. Compared to the currently available PIM-1 inhibitors, the four selected compounds exhibit low Tanimoto coefficient (Tc) similarities, highlighting their structural novelty and druggability. Moreover, all these compounds were found to have a similar chemical boundary (Figure 5). Therefore, models constructed using these selected descriptors have good interpretability and reliability.

Molecular docking studies were conducted to analyze the binding mode of inhibitors at the PIM-1 catalytic domain. Notably, these inhibitors are positioned in the active site, between the residues Leu44, Gly45, Phe49, Lys67, Ile104, Lys67, Leu172, Leu174, and Asp186 (Table 3). These inhibitors were found to have stabilized the complex with hydrogen and hydrophobic interactions with residues, namely, Lys67 and Asp186. This is consistent with earlier research that revealed that these amino acid residues were essential for the catalytic activity of PIM-1 kinase (Qian et al., 2005; Banaganapalli et al., 2016; Shaik et al., 2021; Bima et al., 2022; Shaik et al., 2022).

Although molecular docking has strong computational capabilities, its predictions of the shape of the protein–ligand binding are frequently inaccurate. Thus, in this study, we performed 100-ns MD simulations to test the stability of the chosen compounds in the PIM-1 binding pocket. It was determined that selected compounds remained stable in the binding pocket, as analyzed through the RMSD, RMSF, and hydrogen bonds. Most notably, stable hydrogen bonds with the residues Lys67 and Asp186 were observed in the complexes with the compounds (namely, CHEMBL748285, and CHEMBL690270).

## Conclusion

The PIM kinase family has become a focus of attention in drug discovery. In particular, the search for inhibitors simultaneously targeting PIM-1 isoforms is of great interest because it opens new horizons toward the discovery of new chemicals capable of therapeutically modulating many biochemical pathways involved in the emergence and development of various cancers. In the present study, ensemble learning based on four different machine learning approaches, together with molecular docking and molecular dynamics simulation, was successfully utilized to identify novel scaffold inhibitors against PIM kinase. By combining machine learning and structure-based approaches, it was possible to evaluate the quantitative contributions of the molecules to the activity. This permitted the guided design of four new molecules, predicted to be potential PIM-1 inhibitors. The molecular docking analyses showed that the active inhibitors were able to interact with the amino acids (Lys67, Asp186, Leu44, Glu171, etc.) crucial for catalytic activity of PIM kinase. The interactions were found to be stable, as investigated through 100-ns molecular dynamics simulation.

## Data Availability

The original contributions presented in the study are included in the article/Supplementary Material; further inquiries can be directed to the corresponding authors.
