# Supplementary figures and images for: Combining machine learning and structure-based approaches to develop oncogene PIM kinase inhibitors
Source: Front Chem. 2023 Mar 10;11:1137444. doi: 10.3389/fchem.2023.1137444 (PMC10036574; doi:10.3389/fchem.2023.1137444)

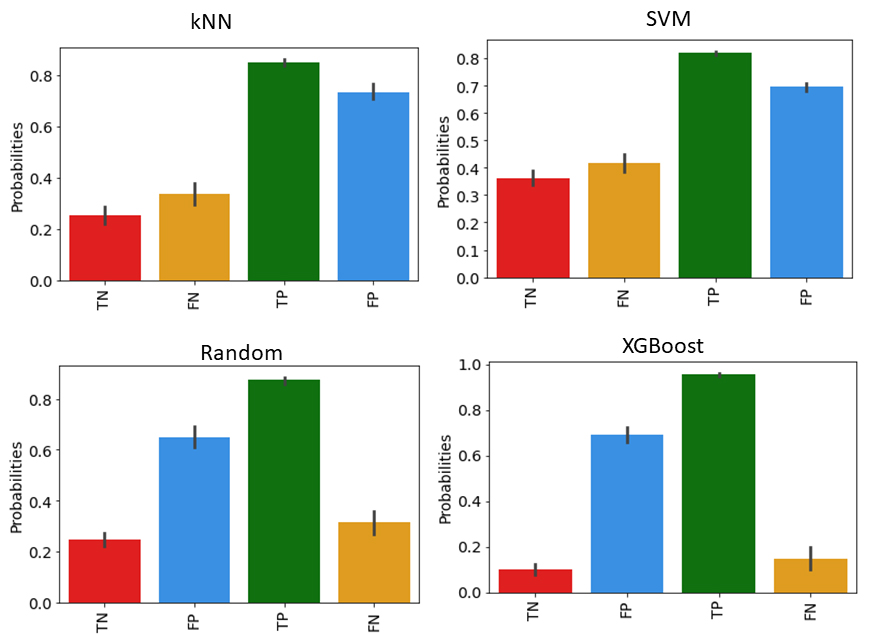

Supplement: Supplementary file 1 [file Image1.JPEG]
