# Supplementary material for: Combining machine learning and structure-based approaches to develop oncogene PIM kinase inhibitors
Source: Front Chem. 2023 Mar 10;11:1137444. doi: 10.3389/fchem.2023.1137444 (PMC10036574; doi:10.3389/fchem.2023.1137444)
Supplement: Supplementary file 2 [file Table1.DOC]

| **Descriptor** | **Descriptor notation** | **Count** |
| --- | --- | --- |
| Path counts | R_TpiPCTPC | 1 |
| Molecular linear free energy relation | MLFER_BH,MLFER_S,MLFER_E | 3 |
| Molecular distance edge | MDEC-33 | 1 |
| Autocorrelation | ATS1m,AATS2i,AATS3i,AATS4i,AATS6s,AATS8s,ATSC4c,ATSC6c,ATSC1v,ATSC4v,ATSC4i,ATSC7i,ATSC8i,ATSC4s,AATSC1c,AATSC7c,AATSC8c,AATSC8i,GATS3c,GATS4c,GATS5c,GATS6m,GATS4e,GATS5e,GATS7e,GATS3i,GATS5i,GATS4s | 28 |
| Information content | SIC3, ZMIC1 | 2 |
| Extended topochemical atom | ETA_Beta_ns_d | 1 |
| Atom type electrotopological state | minHBd,minHBa,minwHBa,minHBint2,mindssC,maxHaaCH  hmin | 7 |
| Atom type electrotopological state | SaasC | 1 |
| Carbon types | C1SP2 | 1 |
| Burden modified eigenvalues | SpMax5_Bhm,SpMin1_Bhm,SpMin2_Bhm,SpMin3_Bhm,SpMax1_Bhv,SpMax2_Bhv | 6 |
| Bond count | nBondsD | 1 |
| Barysz matrix | VE1_DzZ,VE1_Dzs | 2 |

**Supplementary Table 3**. Selected Descriptors using Boruta Method.

**Supplementary Table 4.** Distribution of MACCS fingerprints among active and inactive

Compounds.

| **S.No** | **MACCS** | **Description** | **Active (%)** | **Inactive (%)** |
| --- | --- | --- | --- | --- |
| 1 | MACCS16 | QAA@1 | 68.28 | 18.53 |
| 2 | MACCS38 | NC(C)N | 50.23 | 48.69 |
| 3 | MACCS52 | NN | 47.90 | 33.67 |
| 4 | MACCS62 | A$A!A$A | 73.9 | 91.23 |
| 5 | MACCS65 | C%N | 80.49 | 95.13 |
| 6 | MACCS75 | A!N$A | 42.58 | 35.21 |
| 7 | MACCS82 | ACH2QH | 37.68 | 43.14 |
| 8 | MACCS86 | CH2QCH2 | 39.79 | 40.52 |
| 9 | MACCS92 | OC(N)C | 41.34 | 69.14 |
| 10 | MACCS95 | NAAO | 35.48 | 51.92 |
| 11 | MACCS98 | QAAAAA@1 | 77.27 | 93.72 |
| 12 | MACCS106 | QA(Q)Q | 43.98 | 38.09 |
| 13 | MACCS107 | XA(A)A | 42.37 | 59.21 |
| 14 | MACCS113 | Onot%A%A | 33.72 | 26.37 |
| 16 | MACCS118 | ACH2CH2A > 1 | 50.87 | 60.88 |
| 18 | MACCS131 | QH > 1 | 58.35 | 85.27 |
| 19 | MACCS133 | $A!N | 71.11 | 78.80 |
| 20 | MACCS142 | N > 1 | 89.73 | 99.16 |
| 21 | MACCS144 | Anot%A%Anot%A | 36.95 | 40.65 |
| 22 | MACCS151 | NH | 86.07 | 96.99 |
